# Examining the links between burnout and suicidal ideation in diverse occupations

**DOI:** 10.3389/fpubh.2023.1243920

**Published:** 2023-09-07

**Authors:** Dae Jong Oh, Young Chul Shin, Kang-Seob Oh, Dong-Won Shin, Sang-Won Jeon, Sung Joon Cho

**Affiliations:** ^1^Workplace Mental Health Institute, Kangbuk Samsung Hospital, Sungkyunkwan University School of Medicine, Seoul, Republic of Korea; ^2^Department of Psychiatry, Kangbuk Samsung Hospital, Sungkyunkwan University School of Medicine, Seoul, Republic of Korea

**Keywords:** burnout, exhaustion, suicidal ideation, suicide, depression, occupational health

## Abstract

**Introduction:**

It is uncertain whether burnout is associated with suicidal ideation among workers not in health care services. The aim of this study was to identify how burnout and suicidal ideation are linked among employees in various occupations and whether depression affects this link.

**Methods:**

This cross-sectional study collected data from 12,083 participants aged 19–65 years from 25 companies and public institutions who underwent workplace mental health screening. Burnout and depression were assessed using both the Oldenburg Burnout Inventory and the Center for Epidemiologic Studies Depression Scale. Suicidal ideation was assessed by a self-rated questionnaire from the Korea National Health and Nutrition Examination Survey.

**Results:**

Exhaustion but not the cynicism dimension of burnout was associated with increased odds of suicidal ideation after adjustment for depression and other covariates (odds ratio [OR] = 1.47, 95% CI = 1.26–1.72). The association of exhaustion with suicidal ideation was significant in both depressed (OR = 1.36, 95% CI = 1.14–1.61) and not depressed (OR = 1.77, 95% CI = 1.13–2.76) participants. In exhausted participants, insufficient job control, an unfavorable occupational climate, low educational level, and depression were associated with increased odds of suicidal ideation.

**Conclusion:**

Exhaustion is linked with risk of suicidal ideation in employees not in health care service, regardless of depression status. Exhausted employees, particularly those having poor job resources, should be recognized as an at-risk group.

## 1. Introduction

Suicide is one of the major preventable causes of mortality (1). Globally, most suicide deaths occur in the working-age population 15–64 years old (2). As workplace stress is known to significantly increase the risk of suicide (3), there is an urgent need to identify at-risk employees with occupation-related risk factors for establishing suicide prevention strategies in working individuals.

Burnout, an occupational syndrome of exhaustion, cynicism, and professional efficacy caused by chronic workplace stress (4), has been gaining interest as a potential risk factor for suicidal ideation among employees. All previous works studying the association between burnout and suicidal ideation restricted the study population to health care workers or medical students (5–14). This is probably due to emergence of the concept of burnout from workers in caregiving human services (15). However, burnout is also highly prevalent in workers not in the health care service industry (16), and suicide rates among workers in certain industries, such as construction, transportation, installation, arts, entertainment, and protective services, are higher than those among health care workers (3). Therefore, research is needed to identify whether burnout is associated with suicidal ideation among workers in various occupations.

Based on the complex nature of suicide, addressing burnout and its relationship with suicidal ideation requires understanding of the mediating effects of various confounders. Depression, a strong risk factor of suicidality, should be particularly accounted for in exploring the burnout-suicidal ideation linkage, because burnout could be correlated or overlapping with depressive symptomatology (17), despite recent data supporting the conceptual distinction between burnout and depression (10, 15). Except for one study conducted on North American psychiatrists (13), all previous studies regarding the burnout-suicidal ideation association in health care professionals or medical students did not account for the effect of depression (5, 7–10, 14) or assessed the presence of depression using a brief screening tool consisting of two to four items (6, 11, 12). Such tools have less specificity and reliability compared to standard assessment tools in common use (18, 19). Therefore, it is unclear whether the association of burnout with suicidal ideation is attributable to burnout *per se* rather than to depression. In addition, the associations between burnout, depression, and suicidal ideation have not been explored in workers outside of health care services.

The current study aimed to investigate whether burnout is significantly associated with suicidal ideation among workers in various occupations. This study also aimed to clarify whether the association between burnout and suicidal ideation differs in the presence of depression.

## 2. Materials and methods

### 2.1. Study design, setting, and participants

This cross-sectional study collected data from participants aged 19–65 years who voluntarily underwent workplace mental health screening conducted by the Workplace Mental Health Institute of Kangbuk Samsung Hospital (Seoul, Republic of Korea) from April 2020 to October 2022 upon invitation from their companies. Among the 13,024 respondents, we excluded 941 who did not respond to the assessment for burnout (*n* = 26) or depression/suicidal ideation (*n* = 915). Finally, 12,083 participants were included in the analysis. Participants were employees working in 25 large companies or public institutions classified into industrial divisions based on the Standard Industrial Classifications (20) as follows: two companies in the field of manufacturing (*n* = 4,485); five companies in the field of finance, insurance, and real estate (*n* = 2,925); six public administrations (*n* = 2,829), eight companies in the field of services (941 for accounting and auditing, 181 for amusement and recreation, 322 for health, and 157 for management consultation), three companies in the field of wholesale trade (*n* = 193), and one company in the field of construction (*n* = 50). Supplementary Table 1 shows the descriptive statistics for the participants.

The study was approved by the Institutional Review Board of the Kangbuk Samsung Hospital (KBSMC 2022-03-046), which disregarded the need for informed consent because this study used unidentifiable data collected by routine health screening visits.

### 2.2. Assessments

Burnout was assessed using the Oldenburg Burnout Inventory (OLBI), a 16-item self-rated questionnaire that has been widely used to measure burnout in a variety of occupations (21). The OLBI measures two core dimensions of burnout; eight items for exhaustion and eight items for cynicism or disengagement. Each item is scored using a five-point Likert scale from strongly disagree (1) to strongly agree (5) for a negatively formulated item and reverse scoring for a positively formulated item. We defined the presence of burnout, exhaustion, and cynicism as respective OLBI total, exhaustion, and cynicism scores higher than the sum of the mean and one standard deviation of the total participants (22). The cutoff values for the OLBI scores are shown in Supplementary Table 2.

Suicidal ideation was assessed by a two-item self-rated questionnaire accepted from the Korea National Health and Nutrition Examination Survey (23). Participants were asked to answer the following questions: “Over the past one year, have you ever thought about suicide seriously?” and “Over the past one year, have you planned suicide?” We defined the presence of suicidal ideation as any thought or plan for suicide in the previous year.

Depression was assessed by the Center for Epidemiologic Studies Depression Scale (CES-D), a widely utilized tool validated in various clinical settings, including the community and primary care (24, 25). The CES-D consists of 20 items evaluating the presence and frequency of depressive symptoms over the past week. Each item was scored using a four-point Likert scale from rarely or none of the time (0) to all of the time (3). According to previous studies (24, 25), we defined the presence of clinically significant depression as a CES-D score of 16 points or higher.

Sociodemographic factors, such as age, gender, marital status, education level, and monthly income, were assessed by self-reported questionnaires. Occupation-related factors such as working hours per week, job duration, and occupational stress were also assessed. Occupational stress was measured using the Korean Occupational Stress Scale-Short Form (KOSS-SF), a validated self-reported questionnaire standardized to estimate occupational stress among Korean employees (26). The KOSS-SF includes seven subscales of high job demand, insufficient job control, interpersonal conflict, high job insecurity, dysfunctional organizational system, lack of reward, and unfavorable occupational climate. Each item was rated on a four-point Likert scale from strongly disagree (1) to strongly agree (4). We defined the presence of occupational stress as total or individual item KOSS-SF scores higher than the upper 25th percentile of total participants (Supplementary Table 2) (26).

### 2.3. Statistical analysis

We compared the categorical variables using Pearson’s chi-square tests and continuous variables using Student’s *t*-tests. We conducted stepwise logistic regression analyses to analyze the association between burnout and suicidal ideation. The first model was univariate, the second was adjusted for depression (CES-D score), the third was additionally adjusted for occupational factors (working hours, job duration, and total occupational stress), and the fourth was additionally adjusted for sociodemographic factors (age, gender, marital status, education, and income). The analyses for total burnout and each dimension were performed separately. In analyses by dimension of burnout, exhaustion and cynicism were assessed simultaneously. The burnout * depression interaction terms were also entered into the fully adjusted model to analyze the association of the burnout-depression interaction with suicidal ideation.

To identify whether the association of burnout with suicidal ideation differs in the presence of clinically significant depression, we performed multivariate logistic regression analyses adjusting age, gender, marital status, education, income, working hours, job duration, occupational stress, and CES-D score after stratification of participants by CES-D score ≥ 16 or < 16 points.

To explore the factors associated with suicidal ideation among participants with burnout, we performed multivariate logistic regression with backward elimination for age, gender, marital status, education, income, working hours, job duration, occupational stress, and depression to identify a model with the best fit. To identify which occupation-related stressors were specifically associated with suicidal ideation in employees with burnout, we specified types of occupational stress and entered them into the multivariate model simultaneously.

All statistical analyses were performed using IBM SPSS Statistics, version 19.0 (IBM Corporation).

## 3. Results

Table 1 compares the characteristics of participants by the presence of burnout. Participants with burnout were younger, more likely to be women and unmarried or widowed, and had low education level, low income, and high occupational stress compared to those without burnout. Regardless of the dimensions of burnout, participants having burnout were more likely to have depression and suicidal ideation (Table 1).

**Table 1:** Characteristics of participants by the presence of burnout.

	Burnout, total			Burnout, by dimensions						
	No burnout group (*n* = 10,061)	Burnout group (*n* = 2,022)	*p* ^*^	No exhaustion group (*n* = 9,954)	Exhaustion group (*n* = 2,129)	*p* ^*^	No cynicism group (*n* = 10,083)	Cynicism group (*n* = 2,000)	*p* ^*^
Age, mean (SD)	36.8 (9.4)	33.9 (7.4)	<0.001	36.8 (9.5)	34.1 (7.3)	<0.001	36.9 (9.4)	33.5 (7.3)	<0.001
Women, *n* (%)	2,922 (29.0)	1,102 (54.5)	<0.001	2,891 (29.0)	1,133 (53.2)	<0.001	2,939 (29.1)	1,085 (54.3)	<0.001
Unmarried or widowed, *n* (%)	4,223 (42.8)	1,174 (58.9)	<0.001	4,217 (43.1)	1,180 (56.4)	<0.001	4,214 (42.6)	1,183 (60.1)	<0.001
Low education level, *n* (%)^a^	1,251 (12.7)	149 (7.5)	<0.001	1,240 (12.7)	160 (7.6)	<0.001	1,256 (12.7)	144 (7.3)	<0.001
Low income, *n* (%)^b^	5,086 (54.9)	1,330 (70.4)	<0.001	5,088 (55.5)	1,328 (66.8)	<0.001	5,059 (54.5)	1,357 (72.5)	<0.001
Job duration, years, mean (SD)	10.2 (9.3)	8.1 (7.3)	<0.001	10.2 (9.3)	8.1 (7.2)	<0.001	10.2 (9.3)	7.7 (7.1)	<0.001
Working hours per week, mean (SD)	46.5 (7.2)	48.9 (7.9)	<0.001	46.5 (7.2)	49.2 (7.7)	<0.001	46.6 (7.3)	48.5 (7.7)	<0.001
KOSS-SF score, mean (SD)	38.0 (12.1)	56.6 (11.6)	<0.001	38.2 (12.4)	54.7 (12.2)	<0.001	38.0 (12.1)	56.6 (11.7)	<0.001
CES-D score, mean (SD)	12.4 (8.2)	26.6 (9.9)	<0.001	12.2 (8.1)	26.6 (9.7)	<0.001	12.7 (8.6)	25.1 (10.4)	<0.001
Suicidal ideation, *n* (%)	1,016 (10.1)	713 (35.3)	<0.001	964 (9.7)	765 (35.9)	<0.001	1,087 (10.8)	642 (32.1)	<0.001

KOSS-SF, Korean Occupational Stress Scale-Short Form; CES-D, Center for Epidemiologic Studies Depression scale.

* *p* values from Student’s *t*-test for continuous variables and chi-square test for categorical variables.

a Educated 12 or fewer years.

b Less than 4,000 dollars per month.

Table 2 shows the results from stepwise logistic regression analyses for the association between burnout and suicidal ideation. In the unadjusted model, participants with burnout had almost five times higher odds of suicidal ideation (odds ratio [OR] = 4.85, 95% confidence interval [CI] = 4.34–4.52, *p* < 0.001) compared to those without burnout. The association between burnout and suicidal ideation remained significant even after successive adjustment of covariates including depression. When we conducted logistic regression analyses separating the dimensions of burnout, exhaustion was associated with 47% increased odds of suicidal ideation in the fully adjusted model (OR = 1.47, 95% CI = 1.26–1.72, *p* < 0.001). Cynicism, the other dimension of burnout, was associated with suicidal ideation before but not after adjusting for covariates including depression (Table 2).

**Table 2:** Stepwise logistic regression analyses for association between burnout and suicidal ideation.

	Odds ratio (95% confidence interval)			
	Model 1^a^	Model 2^b^	Model 3^c^	Model 4^d^
Burnout, total	**4.85 (4.34–5.42)**	**1.31 (1.14–1.49)**	**1.30 (1.12–1.51)**	**1.33 (1.14–1.55)** ^*^
Exhaustion	**3.79 (3.32–4.34)**	**1.37 (1.19–1.59)**	**1.40 (1.21–1.62)**	**1.47 (1.26–1.72)** ^**†**^
Cynicism	**1.82 (1.58–2.09)**	1.06 (0.92–1.23)	1.05 (0.90–1.23)	1.01 (0.86–1.19)^**†**^

The analyses for total burnout and dimensions of burnout were performed separately. In analyses for dimensions of burnout, exhaustion and cynicism were entered in the models simultaneously. All the odds ratios with *p* values less than 0.05 are presented in bold.

a Univariate models.

b Adjusted for Center for Epidemiologic Studies Depression scale score.

c Model b + working hours, job duration, and occupational stress.

d Model c + age, sex, marital status, education, and income.

* Akaike information criterion for the model fit = 4359.4.

† Akaike information criterion for the model fit = 3889.0.

When we additionally adjusted for the workplace, the association of exhaustion with suicidal ideation remained significant (OR = 1.40, 95% CI = 1.20–1.63, *p* < 0.001) whereas the association of cynicism with suicidal ideation remained non-significant (OR = 1.00, 95% CI = 0.85–1.18, *p* = 0.966). When we entered the burnout-depression interaction term into the fully adjusted model, the exhaustion * depression interaction was associated with suicidal ideation with marginal significance (OR = 1.37, 95% CI = 0.96–1.95, *p* = 0.079) but the cynicism * depression interaction was not (OR = 1.28, 95% CI = 0.90–1.81, *p* = 0.168).

A total of 320 (4.4%) among the 7,211 participants without depression had suicidal ideation, whereas 1,409 (28.9%) among the 4,872 participants with depression had suicidal ideation. As shown in Table 3, exhaustion was associated with 77 and 36% increased odds of suicidal ideation among the not-depressed and depressed participants, respectively. However, cynicism had no significant association with suicidal ideation, regardless of the presence of depression (Table 3).

**Table 3:** Multivariate logistic regression analyses for the associations of exhaustion, cynicism, and suicidal ideation with depression.^†^

	Number (%) of cases	Adjusted odds ratio (95% CI)	*p*
Not depressed (*n* = 7,211)^a^			
Exhaustion (−)	288/6941 (4.1)	1 [reference]	-
Exhaustion (+)	32/270 (11.9)	1.77 (1.13–2.76)	0.013
Cynicism (−)	286/6836 (4.2)	1 [reference]	-
Cynicism (+)	34/375 (9.1)	1.08 (0.69–1.69)	0.743
Depressed (*n* = 4,872)^b^			
Exhaustion (−)	676/3013 (22.4)	1 [reference]	-
Exhaustion (+)	733/1859 (39.4)	1.36 (1.14–1.61)	<0.001
Cynicism (−)	801/3247 (24.7)	1 [reference]	-
Cynicism (+)	608/1625 (37.4)	0.99 (0.83–1.19)	0.927

Models were adjusted for age, sex, marital status, education, income, working hours, job duration, occupational stress, and Center for Epidemiologic Studies Depression scale (CES-D) score. Exhaustion and cynicism were entered in the models simultaneously.

† Participants with CES-D scores of 16 points or higher were defined as having depression, and those with CES-D scores less than 16 points were defined as not having depression.

a Akaike information criterion for the model fit = 1165.1.

b Akaike information criterion for the model fit = 3174.1.

Table 4 shows the factors associated with suicidal ideation among the 2,129 participants with exhaustion. Insufficient job control, unfavorable occupational climate, and low educational level were associated with increased odds of suicidal ideation in exhausted participants. Depression was associated with more than 4-fold increased odds of suicidal ideation in participants with exhaustion. However, cynicism had no significant association with suicidal ideation. Exhausted participants aged 45 years or older, women, and unmarried or widowed were likely to have suicidal ideation with marginal statistical significance (Table 4). The ORs for the insufficient job control (OR = 1.23, 95% CI = 1.02–1.50, *p* = 0.033), unfavorable occupational climate (OR = 1.32, 95% CI = 1.07–1.63, *p* = 0.009), low education level (OR = 2.12, 95% CI = 1.47–3.08, *p* = < 0.001), and depression (OR = 4.25, 95% CI = 2.87–6.30, *p* < 0.001) remained significant even in the additional adjustment for the workplace.

**Table 4:** Associated factors of suicidal ideation among participants with exhaustion.

	Univariate analysis		Multivariate analysis^†^	
	OR (95% CI)	*p*	OR (95% CI)	*p*
Age ≥ 45 years	1.28 (0.96–1.70)	0.093	1.35 (0.99–1.84)	0.059
Women	1.29 (1.08–1.54)	0.005	1.19 (0.98–1.44)	0.079
Unmarried or widowed	1.25 (1.04–1.49)	0.017	1.19 (0.98–1.45)	0.087
Low education level^a^	1.37 (0.99–1.89)	0.061	1.60 (1.12–2.30)	0.010
Low income^b^	1.12 (0.92–1.36)	0.266	-	-
Job duration < 1 years	0.89 (0.59–1.33)	0.558	-	
Working hours ≥ 40 h/week	0.83 (0.48–1.41)	0.483	-	-
High job demand	0.95 (0.78–1.14)	0.554	-	-
Insufficient job control	1.37 (1.15–1.64)	<0.001	1.24 (1.02–1.50)	0.031
Interpersonal conflict	1.33 (1.11–1.59)	0.002	-	-
High job insecurity	1.28 (1.05–1.55)	0.014	-	-
Dysfunctional organizational system	1.45 (1.20–1.74)	<0.001	-	-
Lack of reward	1.52 (1.22–1.89)	<0.001	-	-
Unfavorable occupational climate	1.52 (1.26–1.82)	<0.001	1.35 (1.11–1.64)	0.003
Cynicism	1.46 (1.22–1.76)	<0.001	-	-
Depression	4.84 (3.31–7.08)	<0.001	4.24 (2.87–6.28)	<0.001

† Results from multivariate logistic regression with backward elimination by entering all the variables of univariate analyses; Akaike information criterion for the model fit = 320.6.

a Educated 12 or fewer years.

b Less than 4,000 dollars per month.

## 4. Discussion

This cross-sectional study demonstrated that the exhaustion dimension of burnout but not the cynicism dimension was associated with suicidal ideation in employees. The association between exhaustion and suicidal ideation was significant in both depressed and non-depressed employees. To our knowledge, this is the first study on the burnout-suicidality linkage among employees in various occupations.

This study found distinct relationships of exhaustion and cynicism with suicidal ideation in employees. Although exhaustion and cynicism often share a common trigger of chronic exposure to occupational stress, these two dimensions of burnout have been acknowledged as distinct conditions (21, 27). Compared to cynicism, exhaustion is more similar to typical stress reactions to excessive job demands, such as fatigue, anxiety, and psychosomatic symptoms, and more predictive of stress-related adverse health outcomes (15, 28, 29). Our findings indicate that exhaustion, compared to cynicism, may be more applicable as a state marker of a stress reaction indicating serious occupation-related stress and potential risk of suicidality.

The results from this study suggest that the relationship between cynicism or disengagement and suicidal ideation could be more complex than that of exhaustion. Conceptual models for burnout have characterized cynicism as a withdrawal or psychological distancing mechanism for defensive coping against exhaustion (15, 29). Therefore, high cynicism in workers may reflect a state of successful compensation against exhaustion in some cases or as a state of excessive exhaustion leading to adverse outcomes in other cases, which might have reduced the apparent significant association between cynicism and suicidal ideation in this study. Consistent with this interpretation, previous studies of health care workers have found mixed results; a negative cynicism-suicidal ideation association in a cross-sectional study of French general practitioners (10) and positive associations in other cross-sectional studies (9, 12, 14) and one longitudinal study (6). A further large-sized study with a prospective design is needed to clarify the impact of cynicism on future risk of suicide and suicidality among workers in a wide range of industries.

This study found that exhaustion was moderately affected by depression but significantly associated with suicidal ideation even after adjustment for depression. Consistent with our findings, a large cross-sectional study of United States surgeons found an independent association of burnout with suicidal ideation (12). However, other studies found no significant association between burnout and suicidal ideation after adjustment for depression (6, 11, 13). These inconsistent findings might be partially attributable to the limited assessment tools for depression, such as the small numbers of self-rated items (6, 11, 12) and a large proportion of items conceptually overlapped with exhaustion (13). Additionally, many studies (6, 11, 12) assessed burnout using the Maslach Burnout Inventory focusing on affective, or emotional, exhaustion rather than physical and cognitive exhaustion (21), which could lead to potential overlap with the assessment of mood status. In the present study, exhaustion increased the odds of suicidal ideation by 77% for not depressed employees, but that odds decreased to 36% for depressed employees. As depression is one of the strong risk factors for suicidality, the attributable risk of suicidality to burnout may be attenuated by the increasing proportion of depression in the study population. Therefore, the difference in proportions of participants with depression over a clinically significant threshold also might impact the inconsistent findings among studies.

The crucial finding of this study is that exhaustion is significantly associated with suicidal ideation in employees even in the absence of depression. Although depression is one of the most crucial risk factors of suicide, a substantial proportion of death by suicide occurs in the general population without depression (30–33). Therefore, exhaustion can be a promising target for establishing prevention strategies against suicide in non-depressive adults of working age. We found that exhausted employees with insufficient job control and unfavorable occupational climate could be an at-risk group for suicidality. The association between low educational level and suicidal ideation might also converge with lack of job control, as attaining a higher position with sufficient autonomy generally requires a higher educational level. Our findings suggest that sufficient job resources for adapting to excessive job demands could be crucial to attenuate the risk of suicidality in the workplace. Previous studies have reported that job control may attenuate the negative effect of mental strain and improve the general mental health of employees (34–36). Encouragement of autonomous decision making, creativity, and exhibition of skills and changes into a non-authoritative, non-discriminative, and predictive job climate could be helpful to reduce the risk of suicidality of exhausted employees in the workplace.

Several limitations should be acknowledged in this study. First, this study could not verify the causal relationship between burnout and suicidal ideation due to the cross-sectional design. A further prospective study is needed to elucidate whether burnout imposes the risk of suicide on employees. Second, generalization of our findings is limited because this study only included data from workers whose companies or institutions requested the mental health examinations. Selection bias could not be excluded as workers from the companies in specific industries, such as manufacturing and finance, comprised the majority of study participants. Our findings should be replicated by further studies of a representative sample of workers. Third, key variables such as depression and suicidal ideation were evaluated by self-reported questionnaires, which could be vulnerable to response bias. Finally, there was no information about therapeutic intervention (e.g., psychotherapy, counseling, or pharmacotherapy) mitigating the symptoms of burnout and suicidality.

Despite these limitations, this large cross-sectional study is the first to identify the association between burnout, depression, and suicidal ideation in employees from a variety of occupational areas. Burnout, especially exhaustion, is worth being considered in the risk assessment of suicidality in workers. This may be particularly necessary when workers have insufficient job resources. Future investigations are warranted to determine whether timely screening and interventions for burnout are effective in reducing workplace suicidality.

## Data availability statement

The raw data supporting the conclusions of this article will be made available by the authors, without undue reservation.

## Ethics statement

The studies involving humans were approved by Institutional Review Board of the Kangbuk Samsung Hospital (KBSMC 2022-03-046). The studies were conducted in accordance with the local legislation and institutional requirements. Written informed consent was waived by the Institutional Board because the study used unidentifiable data collected by routine health screening visits.

## Author contributions

DO, YS, SC, and S-WJ conceptualized and designed this work. DO, YS, K-SO, D-WS, S-WJ, and SC acquired and analyzed the data. DO, SC, and S-WJ drafted the manuscript. All authors contributed to the article and approved the submitted version.

## Funding

This work was supported by Medical Research Funds from Kangbuk Samsung Hospital. The sponsor was not involved in the design or administration of the study; collection, management, analysis, or interpretation of the data; preparation, review, or approval of the manuscript; or decision to submit the manuscript for publication. S-WJ and SC had full access to all data in the study and had final responsibility for the decision to submit it for publication.

## Conflict of interest

The authors declare that the research was conducted in the absence of any commercial or financial relationships that could be construed as a potential conflict of interest.

## Publisher’s note

All claims expressed in this article are solely those of the authors and do not necessarily represent those of their affiliated organizations, or those of the publisher, the editors and the reviewers. Any product that may be evaluated in this article, or claim that may be made by its manufacturer, is not guaranteed or endorsed by the publisher.

## Supplementary material

SUPPLEMENTARY Table 1 Characteristics of the total participants.

SUPPLEMENTARY TABLE 2 Cutoff values for burnout and occupational stress.
