# Supplementary material for: Examining the links between burnout and suicidal ideation in diverse occupations
Source: Front Public Health. 2023 Sep 7;11:1243920. doi: 10.3389/fpubh.2023.1243920 (PMC10513409; doi:10.3389/fpubh.2023.1243920)
Supplement: SUPPLEMENTARY Table 1 — Characteristics of the total participants. [file Table_1.DOC]

**Supplementary Table 1. Characteristics of the total participants**

|  | Total  participants  (n = 12,083) |
| --- | --- |
| Age, mean (SD) | 36.3 (9.2) |
| Women, n (%) | 4024 (33.3) |
| Unmarried or widowed, n (%) | 5397 (44.7) |
| Low education level, n (%)^a^ | 1400 (11.6) |
| Low income, n (%)^b^ | 6416 (53.1) |
| Job duration, years, mean (SD) | 9.8 (9.0) |
| Working hours per weeks, mean (SD) | 46.9 (7.4) |
| KOSS-SF score, mean (SD) | 41.1 (13.9) |
| CES-D score, mean (SD) | 14.7 (10.0) |
| Suicidal ideation, n (%) | 1729 (14.3) |

KOSS-SF, Korean Occupational Stress Scale-Short Form; CES-D, Center for Epidemiologic Studies Depression scale

a. Educated 12 or fewer years

b. Less than 4000 dollars per month

**Supplementary Table 2. Cutoff values for burnout and occupational stress**

|  | Cutoff values (points) |
| --- | --- |
| OLBI scores for burnout^a^ |  |
| Burnout, total | 47.50 + 11.48 |
| Exhaustion | 23.83 + 6.21 |
| Cynicism | 23.67 + 5.94 |
| KOSS-SF scores for occupational stress^b^ |  |
| Total score | 49.6 |
| High job demand | 66.67 |
| Insufficient job control | 58.33 |
| Interpersonal conflict | 44.44 |
| High job insecurity | 33.33 |
| Dysfunctional organizational system | 58.33 |
| Lack of reward | 55.56 |
| Unfavorable occupational climate | 50.0 |

OLBI, Oldenburg Burnout Inventory; KOSS-SF, Korean Occupational Stress Scale-Short Form

a. Cutoff values were calculated as the sum of scores of the mean and one standard deviation.

b. Cutoff values were defined as the standardized scores of the upper 25^th^ percentile of total participants (n = 12,083).
